# Dietary Polyphenols: A Multifactorial Strategy to Target Alzheimer’s Disease

**DOI:** 10.3390/ijms20205090

**Published:** 2019-10-14

**Authors:** Sudip Dhakal, Naufal Kushairi, Chia Wei Phan, Benu Adhikari, Vikineswary Sabaratnam, Ian Macreadie

**Affiliations:** 1 School of Science, RMIT University, Bundoora, Victoria 3083, Australia; sudip.dhakal@rmit.edu.au (S.D.); benu.adhikari@rmit.edu.au (B.A.); 2 Mushroom Research Centre, University of Malaya, 50603 Kuala Lumpur, Malaysia; naufal.kushairi@gmail.com (N.K.); phancw@um.edu.my (C.W.P.); viki@um.edu.my (V.S.); 3 Department of Anatomy, Faculty of Medicine, University of Malaya, 50603 Kuala Lumpur, Malaysia; 4 Department of Pharmaceutical Life Sciences, Faculty of Pharmacy, University of Malaya, 50603 Kuala Lumpur, Malaysia; 5 Institute of Biological Sciences, Faculty of Science, University of Malaya, 50603 Kuala Lumpur, Malaysia

**Keywords:** Alzheimer’s Disease, amyloid beta, antioxidant, longevity, mushroom, neuroprotection, nutraceuticals, protein homeostasis, polyphenol

## Abstract

Ageing is an inevitable fundamental process for people and is their greatest risk factor for neurodegenerative disease. The ageing processes bring changes in cells that can drive the organisms to experience loss of nutrient sensing, disrupted cellular functions, increased oxidative stress, loss of cellular homeostasis, genomic instability, accumulation of misfolded protein, impaired cellular defenses and telomere shortening. Perturbation of these vital cellular processes in neuronal cells can lead to life threatening neurological disorders like Alzheimer’s Disease, Parkinson’s Disease, Huntington’s Disease, Lewy body dementia, etc. Alzheimer’s Disease is the most frequent cause of deaths in the elderly population. Various therapeutic molecules have been designed to overcome the social, economic and health care burden caused by Alzheimer’s Disease. Almost all the chemical compounds in clinical practice have been found to treat symptoms only limiting them to palliative care. The reason behind such imperfect drugs may result from the inefficiencies of the current drugs to target the cause of the disease. Here, we review the potential role of antioxidant polyphenolic compounds that could possibly be the most effective preventative strategy against Alzheimer’s Disease.

## 1. Introduction

**Figure 1:**
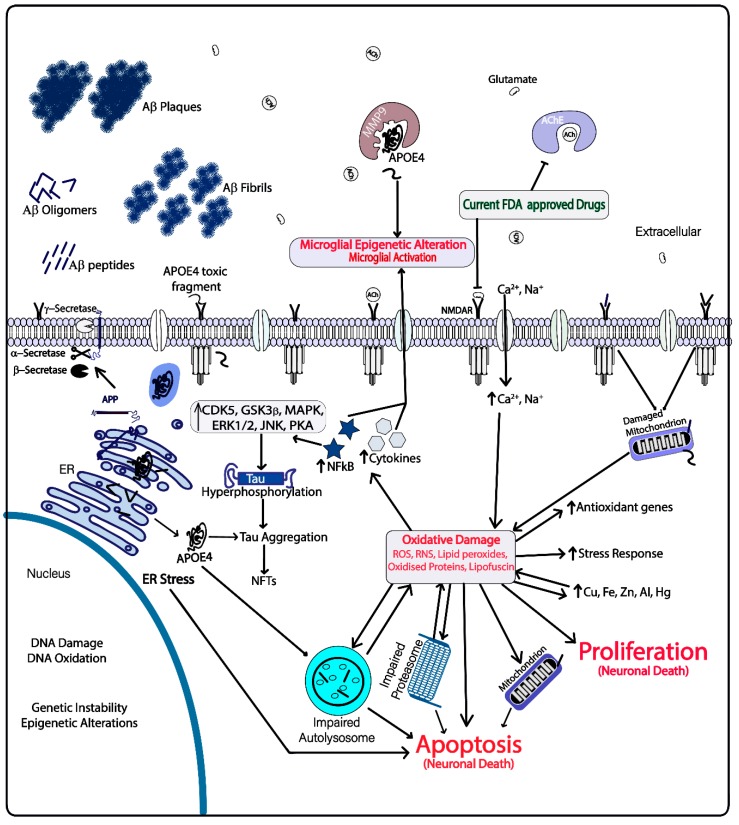
Current drug targets and molecular events occurring in the Alzheimer’s Disease (AD) brain microenvironment.

Deaths due to Alzheimer’s Disease (AD) and other dementias are a major cause of mortality in the elderly worldwide, and the rate is increasing rapidly with a doubling time of 20 years [1]. AD is an age-related neurodegenerative disease that leads to cognitive impairment and death. Neuronal synapsis disruption, accumulation of amyloid plaques in brain, formation of neurofibrillary tangles in neuronal cells, loss of cellular homeostasis and accumulation of oxidative stress are major hallmarks of the disease [2]. However, mitochondrial dysfunction, loss of protein and lipid homeostasis, alterations in biometal distribution, cellular senescence, loss of nutrient sensing and accumulation of misfolded proteins are also associated with the AD [3]. Despite the efforts of more than three decades of research, the precise cause of AD has not been found. Many hypotheses have been made to address the major molecular events in the neuronal cells with AD (refer to Figure 1) [2]. Polyphenolic compounds have been reported to have multiple effects in cells including inducing antioxidant activity, induction of autophagy, restoration of lipid homeostasis, antiproliferative property, anti-proteinopathies, inhibition of choline esterases, anti-inflammatory activity, metal chelation, clearance of lipofuscin and others (refer to Table 1). This review details how polyphenols exert their neuroprotective role at the cellular level helping to prevent and possibly cure AD.

## 2. Current Therapeutic Approaches Only Target Symptoms of AD

Consideration for drug design against AD has come from the symptoms. Traditional approaches based on cholinergic dysfunction have been highly utilised for treatment of AD [2]. Current FDA approved drugs include donepezil, rivastigmine, galantamine and memantine of which the first three drugs are acetylcholine esterase inhibitors, while memantine targets the N-methyl-D-aspartic receptor (NMDAR) [92,93]. Damage of cholinergic neuronal cells leading to the reduced levels of acetylcholine, a neurotransmitter involved in cognition and synapsis, has been found to be associated with AD [94]. Restoring the levels of acetylcholine in an AD brain has been considered to be the most viable palliative measure. The inhibition of acetylcholine esterase has shown benefits in restoring cognition making it a primary care strategy [95]. Likewise, memantine is a NMDAR antagonist as it selectively inhibits the interaction of glutamate with NMDAR, balancing the excitation by the neurotransmitter. The drug effect comes through the reduction of ionotropic channels in the membrane restoring the balanced influx of calcium and sodium ions which is highly expressed in an AD brain causing excitotoxicity [92,96]. However, the strategy targeting only these extracellular events may not provide substantial protection, as many intracellular processes are also altered during progression of AD.

## 3. Therapeutic Strategies Based on Targeting Amyloid β and Tau Proteins

Several studies involving novel strategies to multiple molecular processes, have been considered. The most popular one among the various newer approaches is targeting amyloid β, also referred to as anti-amyloid strategy. Amyloid β comprise short polypeptides, 36–43 amino acid long, produced after pre-processing of amyloid precursor protein (APP) by two different enzymes, namely β-secretase (BACE) and γ-secretase [97,98]. BACE cleaves the APP at a specific site followed by the action of γ-secretase resulting in the formation of peptides of length 36-43 amino acids. The most important polypeptide found in the amyloid plaques of the patient’s brain is Aβ42, which is well-known for its adverse effects in different disease models [99]. Conversely, α-secretase can cleave APP at a site within Aβ, creating shorter fragments also called Aα, which is non-amyloidogenic and protective [100,101]. BACE exists in two isoforms, namely BACE1 and BACE2 [102,103]. BACE1 has been considered an important drug target as it is intimately involved in the formation of Aβ [104]. The BACE1 enzyme has the aspartic catalytic residues located at the interface of the N-terminus and C-terminus forming a dyad, one of which acts as an acid and the other one as a base during the proteolysis [105,106]. The recent developments enlightening BACE1 structure and function provided opportunities for *in silico* molecular docking studies supporting drug design and discovery [107]. Various molecules have been studied and evaluated for their inhibitory action against BACE1 including macrocyclic lactones, hydroethylenes, aminoethylenes, aminoimidazoles, aminobenzthiazines, spiropiperidines, etc. [108,109,110,111,112,113,114].

Inhibition of γ-secretase activity is also an important approach in the anti-amyloid strategy. Inhibiting activity of γ-secretase will affect the Aβ formation and is expected to halt the amyloidogenic progress and associated toxicity. However, the interference with the γ-secretase activity also affects the notch signaling [115]. Development and cellular growth are associated with notch signaling mechanism, which will also be altered by inhibiting the γ-secretase [116]. Considering these side effects of the γ-secretase inhibitors, different sulfones and sulfonamides that do not affect notch signaling have been evaluated for their activity against γ-secretase [117]. An anti Aβ-aggregation approach has also been studied in the effort to find a chemo preventative for AD. Aβ aggregation occurs by the interaction of molecules of monomeric Aβ which further interact with other monomeric forms to produce aggregates [118]. Oligomeric forms of Aβ42 have been reported to be the most toxic species. Very few compounds have been evaluated for their anti-aggregation properties [119,120,121,122]. Aβ clearance, inducing misfolded protein degradation through induction of autophagy and unfolded protein response, is another strategy that could provide protection [123]. Furthermore, vaccines and antibodies against Aβ were also evaluated for their efficacy against AD [120,124,125]. Early vaccines targeting Aβ caused serious side effects of meningoencephalitis in the trial and antibodies are limited by the blood brain barrier as only 0.1% of the antibodies were found to cross it [1]. While Aβ remains an important target, its clearance may have limited benefits as a cure after the disease onset. Despite the limitations of approaches targeting Aβ, early prevention of Aβ formation and its clearance remains a top priority.

Tau neurofibrillary tangles (NFTs) are another important pathophysiological hallmark in addition to the accumulation of the amyloid plaques in the AD brain [126]. In a normal brain, tau protein plays a critical role in cellular integrity by maintaining the microtubules [127]. Tau normally stays in the membrane of axons in phosphorylated form, as it contains 84 amino acid residues where phosphorylation can occur [128]. Hyperphosphorylation of these tau proteins leads to self-interaction and reduces its tendency to bind with the microtubules causing the formation of the NFTs [120,129]. Formation of NFTs is associated with alteration in neuronal plasticity and synapsis [116]. Hyperphosphorylation of tau has been reported to be the major contributor for activation of the microglial cells and astrocytes [130]. Activation of these immunomodulators downstream leads to the release of nuclear factor kappa B (NFκB) and cytokines [131], which cause the brain inflammation associated with AD. Meanwhile, release of the inflammatory mediators like NFκB and interleukins result in the activation of protein kinases in cell, which reinforces the hyperphosphorlation of the tau [132,133]. Some of the important protein kinases reported to cause hyperphosphorylation of tau include mitogen-activated protein kinase (MAPK), cyclin dependent kinase-5, tau protein kinase-I and glycogen synthase kinase-3β (GSK-3β) [120]. Inhibition of these protein kinases, specifically cyclin dependent kinase 5 (CDK5) and GSK-3β, has been evaluated in previous studies as important molecular targets in treating AD. Non-selective CDK5 inhibitors like (R)-roscovitine and (R)-CR8 are still under investigation to provide better understanding of their neuroprotective effect [134,135]. Likewise, different classes of inhibitors of GSK3β such as lithium ions, thiazoles, indirubins, thiadiazolidinones, hymenialdisine and others have been reported for their potential protective effect against AD [136,137,138,139,140]. Additionally, immunological approaches (active and passive immunization) against various forms of tau are areas of increasing research interest. Unlike anti-amyloid antibodies and vaccines, anti-tau vaccine and antibodies are reported to have promising effects against AD [141,142]. These strategies are expected to reduce the formation of tau tangles and help in synapsis and neuronal plasticity.

## 4. Prospect of APOE4 as a Drug Target for AD

Another independent risk factor in AD is apolipoprotein E4 (APOE4) protein, which normally helps in the transportation of the cholesterol through the APOE receptors [143]. Higher expression of APOE4 has been reported to be associated with the late onset of the disease [144]. There is evidence that APOE4 proteins induce Aβ aggregation and reduce Aβ clearance [145]. Furthermore, APOE4 proteins not only target Aβ interaction, but are also linked to tau hyperphosphorylation, energy metabolism and inflammation in neurons [146,147,148]. The inflammatory response in the brain leads to the proteolysis of APOE4 that may lead to the formation of highly bioactive toxic molecules [149]. Formation of these bioactive fragments of the APOE4 disturb the energy metabolism by altering mitochondria. Furthermore, early evidence shows that APOE4 effects are more pronounced in females, implying possible participation of sex hormones such as estrogen in determination of AD progression. Studies to unravel the actual cause of the gender effect could be a guide for novel approaches to prevent AD [150].

## 5. Reactive Oxygen and Reactive Nitrogen Species in AD

Oxidative stress accumulation is an important event during AD that worsens as the disease progresses [151]. Oxidative stress is triggered by the accumulation of free radicals such as reactive oxygen species (ROS) and reactive nitrogen species (RNS) and the inability of the cells to clear these reactive molecules. Formation of these free radicals occurs in the electron transport chain due to the loss of the electrons during transfer in the mitochondrial membrane [152]. Formation of free radicals can occur in neurons by multiple factors including mitochondrial dysfunction, impaired autophagy, disruption of lipid homeostasis, formation of lipofuscin, Aβ-induced oxidative damage and accumulation of transition elements (such as iron, copper, zinc, aluminium and mercury) [152,153,154]. These free radicals can oxidize proteins, lipids and DNA affecting various important metabolic processes in the neuronal cells [153,155]. They can also activate the expression of pro-inflammatory markers, such as NFκB and cytokines, which contributes to the recognition of damaged cells [156]. Clearance of these toxic species (ROS and RNS) and balancing the redox state is a requirement for cells to function normally. Several antioxidant genes are expressed to protect the cells from oxidative damage. Young cells function efficiently to clear these free radicals, whereas older cells are thought to be less efficient in doing so [157]. Accumulation of ROS and RNS for a longer duration puts the cells under chronic oxidative stress and initiates abnormal changes. It is still not clear whether oxidative stress accumulation that occurs during ageing is the cause of AD or the aftermath of the disease progression. No matter what comes first, it is evident that oxidative damage is the detrimental event in AD that kills the neuronal cells [158]. Studies on Aβ42 expressed in yeast show that Aβ42 can cause the mitochondrial dysfunction, enhance stress response and upregulate expression of protective antioxidant genes signifying oxidative stress accumulation [154,159]. Biometals involved in oxidative stress management in the cells include iron, aluminium, mercury, zinc and copper [160]. Altered levels of iron, zinc, copper and aluminium have been reported in AD brains [3,161]. With excessive oxidative stress accumulation, protein degradation by cathepsins in lysosomes may also get impaired due to the formation of oxidizing complex molecules like lipofuscin. This can lead to the impairment of the autophagic clearance. In the meantime, lipofuscin further increases oxidative damage to cells by catalysing the Fenton reaction accelerating formation of free radicals [162].

In early stages of AD, Aβ’s entry in mitochondria disrupts the mitochondrial function and generates free radicals [163]. Additionally, APP and Aβ are also reported to be localized in the membrane of mitochondria thereby disrupting the normal electron transport chain. The disruption causes the loss of electrons from the mitochondrial membrane [164]. In summary, redox dyshomeostasis in cells negatively impacts the cellular processes and metabolism that includes impairments to: protein clearance, mitochondrial function, biometal homeostasis, calcium homeostasis, inflammatory responses and antioxidant capacity [165]. Considering these facts, the search for drugs targeting the early relief from oxidative stress in the neuronal cells could be beneficial for preventing neurodegenerative diseases including AD. Antioxidants extracted from various plants can be a natural source of nutraceuticals and prospective therapeutics. Approaches of antioxidant therapy using natural compounds, such as stilbenes, flavonoids, epicatechin, *Gingko biloba* extracts, ascorbic acid, melatonin and curcumin, have been found to have beneficial effects against AD [166,167,168,169,170,171,172]. Studies have also shown that the reduction of Aβ in neuronal cells can be achieved using antioxidant therapy. In addition, therapies that restore the normal mitochondrial function or mitochondrial regeneration are also found to restore redox balance in cells [158]. Similarly, ongoing studies of metal chelators in combination with other strategies are also considered as a more effective approach [173,174,175].

## 6. Single Target Strategies in Management of AD

New approaches to treat AD have also considered other targets that are associated with progression of AD. Regulating γ-amino butyric acid (GABA) receptors is one such approach, as GABA is produced by decarboxylation of the neurotransmitter glutamate which ultimately affects the excitotoxic pathway [176]. There are two different isoforms of GABA receptors: GABA 1 and GABA 2 [177]. Many compounds targeting both receptors have been studied to assess their effectiveness to treat AD, but none have shown promising results [178,179]. Despite the initial unsuccessful clinical trials, there is room for hope and studies are continuing.

Phosphodiesterase is another drug target used in previous studies. It normally cleaves phosphodiester bonds in the secondary signaling molecules such as cGMP and cAMP, thus affecting the signal transduction [180]. Various phosphodiesterase inhibitors have been studied to assess their neuroprotective effect [181,182,183]. Cyclooxygenases, COX-2 in particular, have also been used as a target for treatment of AD. COX-2 has been reported to induce Aβ42 formation by increasing γ-secretase activity through prostaglandin formation [184]. Furthermore, COX-2 has been found to activate NMDA receptors, thereby causing excitotoxicity in neuronal cells [185]. Recent studies have shown limited involvement of COX-2 in Aβ deposition, however COX inhibitors are still beneficial in treatment of AD [186].

Histaminic receptors (H3) have a role in releasing neurotransmitters such as acetylcholine, dopamine, nor-epinephrine, histamine and serotonin [187]. Interference in functions of these receptors by antagonists has revealed that they have a protective role in tau-associated memory deficits [188]. Serotonergic receptors have also been studied for their role in cognitive dysfunction, amyloid formation and neuroinflammation during progression of AD [189]. Inhibition of these receptors activated neurotransmitters, glutamate and acetylcholine in particular, improving cognition in AD patients and suggesting that these receptors can be important targets for drug development [190].

The peroxisome proliferator-activated receptor γ (PPARγ) is another target in drug development against AD. PPARγ, a nuclear receptor found in restricted brain areas, has important role in glucose and lipid metabolism [191,192]. Furthermore, PPARγ has been demonstrated to enhance neuronal inflammation and damage [193]. Inhibition of these receptors reduced Aβ aggregates and expression of neuroinflammatory mediators [194,195]. Agonists of PPARγ were found to have other functions in AD brains including clearance of Aβ, disaggregation of Aβ plaques, and reduction of APOE4 expression, however severe side effects of these compounds led to the cessation of the clinical trials [196,197,198,199,200].

The endocannabinoid system is another pathway that is targeted for drug development for AD. Targeting different processes in the system has been reported to increase cognition and the anti-inflammatory response. Targeting the endocannabinoid system reduced Aβ-induced toxicity and tau hyperphosphorylation [201]. The system comprises two lipid molecules derived from endogenous arachidonic acid which bind with two different receptors, CB1 and CB2. The binding of these lipids depends on two different hydrolytic enzymes, fatty acid amide hydrolase (FAAH) and monoacylglycerol lipase (MAGL) [202]. Drug designers have considered these molecules, including the receptors and enzymes involved, as potential therapeutic target [203,204,205].

Cholesterol has been considered as one of the risk factors for AD and it has been demonstrated that it also contributes to the formation and accumulation of Aβ [206]. Cholesterol lowering drugs are thus important drugs that show benefits against AD [207]. While some studies of statins and AD are inconclusive, other studies support their neuroprotective role [208,209]. Studies in yeast show that statins reduced the levels of intracellular recombinant Aβ implying the possible induction of autophagy [210].

Neurotrophic factors or neurotrophins (NTs) including nerve growth factor (NGF), brain-derived neurotrophic factor (BDNF), neurotrophin 3 (NT3), and neurotrophin 4 (NT4) are crucial for development, maintenance, repair and survival of neuronal populations [211,212,213]. These polypeptides exert their actions through binding and specifically activating tropomyosin receptor kinases (Trk) of either TrkA, TrkB and TrkC [214]. The activation of the receptors induces phosphorylation of the cytoplasmic domain kinases and stimulates signaling pathways including phosphatidyloinositol-3-kinase (PI3K)/protein kinase B (Akt), mitogen-activated protein kinase (MAPK) and phospholipase C-γ1 [215] which are responsible for survival, growth, neuronal differentiation, neurogenesis and neuroplasticity [214,216].

First discovered by Levi-Montalcini in 1951 [217], NGF was shown to be important in the neuronal plasticity and survival of cholinergic neurons in the cerebral cortex, hippocampus, basal forebrain and hypothalamus [218]. The reduction in NGF amount and activity are substantial in the AD [219,220,221]. Therefore, administration of NGF to targets survival and synaptic functions of cholinergic neurons could be useful in the therapeutic prevention and treatment of the disease [222,223].

Exogenous administration of NGF in animals was found to improve the cholinergic system in the CNS, particularly in the forebrain and hippocampus, leading to enhanced cognitive function [224,225,226,227,228]. However, exogenous administration of NGF to combat AD is a difficulty as this protein does not normally pass through the blood brain barrier (BBB) [229,230,231]. In addition, direct delivery of neurotrophic factors may exert serious peripheral side effects [232]. These limitations have brought about innovations to enhance the bio-delivery of NGF for AD therapy by using stem cells, viral vectors, small molecule modulators and most recently, encapsulated cell biodelivery (ECB) [233]. While these methods are known to be costly, hard to administer and precarious, consumption of bioactive compounds from natural products are increasingly preferred in an attempt to slow down and prevent disease progression.

## 7. Drug Combinations as a Strategy for AD Therapy

Requirements of multifactorial drug design resulted in the development of drug combination strategies. Drugs that target at least two molecular targets of AD were tested for efficacy against AD. A series of hybrid compounds produced by combining two drugs that inhibit AChE and BACE1 has been reported [234,235,236,237,238,239]. Other combinations were found to be effective in metal chelating activity and antioxidant properties with less toxicity [237,239]. Similarly, combinations of AChE with GSK3β inhibitors, MAO inhibitors, metal chelators, NMDAR inhibitors, 5-HT receptor inhibitors, histaminic receptors inhibitors and phosphodiesterase inhibitors have been studied [240]. Some drugs designed in this way have been reported to alleviate AD. However, a number of drug combinations were discontinued due to their adverse effects or low activity [240]. Furthermore, combinations of BACE1 with a GSK3β inhibitor, metal chelators with MAO-B and phosphodiesterase inhibitors were also studied for their efficacy as multitarget therapy [241,242,243,244]. The different combinations of drugs, targeting multiple events of AD pathology hallmarks, may provide substantial protection and possibly cure AD (reviewed in [240]).

## 8. Restoring Protein Homeostasis as a Novel Multifactorial Approach

Disruption of protein homeostasis is one of the major hallmarks of the age-related neurological disorders [245]. There are different mechanisms by which proteostasis is regulated within the cell. The unfolded protein response (UPR), ubiquitin proteasome system and autophagy are responsible for maintaining the protein balance within cells [246]. Impairment in these processes leads to the accumulation of unwanted cytosolic garbage. Ageing normally comes with less efficient cellular processes including proteostasis [247].

The UPR occurs as misfolded proteins start accumulating, causing ER stress. The inositol response element 1 (IRE1), activating transcription factor 6 (ATF6) and PRK-like ER kinase (PERK) proteins play crucial roles in sensing the presence of aberrant proteins and triggering the upregulated expression of chaperones and foldases to rectify protein folding errors. This ultimately takes the aberrant proteins through ER-associated proteasomal degradation. During ageing, the proteins involved in the UPR are expressed in low levels signifying that upregulating the expression of the proteins will be a potential strategy for preventing or slowing down protein misfolding diseases during ageing [248].

During post translational modification of proteins, ubiquitination of lysine residues is normal. This allows the selective degradation of inappropriately folded proteins mitigating their negative effects. Three different enzymes, namely E1 activating/carrier ubiquitin enzyme, E2 and E3 ligase, interact to transfer ubiquitin to the target proteins’ lysine residues. The ubiquitin tags are removed by deubiquitinating enzymes in normal conditions. But in the case of misfolded proteins, the process of ubiquitination continues several times leading to the formation of polyubiquitin tags in the protein, which is recognized by proteasomal receptors for further processing. Proteasomes are found as complexes called 26S complex that contain two subunits (20S catalytic unit and 19S regulatory unit). The catalytic 20S unit is composed of three proteolytic subunit classes β1 (caspase like activity), β2 (trypsin like activity) and β5 (chymotrypsin like activity) [249]. These proteases not only target polyubiquitinated proteins but also degrade the oxidized proteins [246]. This process of tagging the unwanted proteins with polyubiquitin tags and degrading them through the proteasomes is also referred to as the ubiquitin proteasome system.

Clearance of misfolded proteins, damaged organelles and global turnover of the components of the cell takes place through autophagy [250]. Autophagy can be of three different types including microautophagy, chaperone mediated autophagy and macroautophagy [250]. Microautophagy is the normal process of engulfment of unwanted material of the cytosol in the lysosomal vesicle [251]. In the lysosomal vesicle, different enzymatic action degrades the engulfed unwanted material. Chaperone-mediated autophagy is another system which acts through chaperone proteins (heat shock proteins like Hsp70), which initially bind with the misfolded protein and refold it. When the refolding fails, the chaperones drive these bound materials to the lysosomal vesicles through the lysosomal receptor (LAMP2A) for lysosomal degradation [252]. In addition to these local events of protein clearance, a huge turnover of the cellular molecules/organelles occurs through macroautophagy for supply of required components during different stages in the cell cycle [253]. Macroautophagy, also termed as autophagy hereafter, was initially identified as the effect of starvation [254]. Increase in AMP/ATP ratio during starvation activates AMPK and inhibits protein kinase B (Akt)/mechanistic target of rapamycin (mTOR) pathway, activating the initiation of the autophagosome formation. ROS (dihydronicotinamide-adenine dinucleotide phosphate/NADPH oxidase-induced) accumulation, PI3K/Akt/mTOR inhibition, AMPK, Beclin1, transcription factor EB (TFEB) and sirtuin 1 (SIRT1)/fork head box like protein (FOXO) activation are known pathways for inducing autophagy [250,255,256,257,258].

In AD, accumulation of aberrant Aβ is an example of the disruption of protein homeostasis. Disruption of proteostatis is considered to be the major cause of Aβ accumulation. Mitochondrial dysfunction, ROS accumulation, lipid peroxidation and expression of stress response genes are the consequence of Aβ toxicity in cells. Alterations in the redox state, impairment in protein degradation system, altered distribution of biometals, cellular senescence and cell death are the consequences of the impact in neuronal cells [2,3]. Furthermore, generation of lipofuscin due to increased oxidative stress is another part of the story as these highly lipophilic reactive species catalyse the Fenton reaction causing generation of more free hydroxyl radicals. This leads to the irreversible damage of the cells by oxidizing lipids, proteins and DNA [153]. Impairment in lysosomal and proteasomal degradation is also associated with accumulated lipofuscin in these cellular compartments [259]. Lipofuscin is a complex of molecules formed by the combination of lipid peroxides, oxidized proteins, transition metals and some carbohydrates [153]. Disrupted autophagy may also result in the impairment in lipolysis causing the lipid dyshomeostasis in the cells [260,261]. In intracellular environments of dividing cells, lipofuscin is neither digested nor exocytosed, however it is diluted through cell division. Conversely in neuronal cells, lipofuscin aggregates cannot be diluted through cell division as neuronal cells remain in the G_0_ part of the cell cycle. Attempted division of these cells induces cell death [153,262]. The drug that clears lipofuscin from the cell could restore protein homeostasis and possibly cure AD. Overall, protein homeostasis maintenance and redox state balance in cells could provide efficient early intervention and limit the disease progression. Targeting the restoration of protein homeostasis has also been hypothesized to provide protection against various other neurodegenerative diseases.

Restoring the protein balance is believed to protect neuronal cells to overcome age-related changes. Protein dyshomeostasis is considered to be a prime factor of oxidative damage, mitochondrial dysfunction, epigenetic alterations, altered biometal distribution, accumulation of aberrant proteins, aggregation of proteins, lipid dyshomeostasis, altered energy metabolism and cell death during progression of AD. Furthermore, inducing processes like autophagy may even increases synapsis, cognition and longevity of the neuronal cells [263,264]. These multiple effects of restoring protein balance in ageing cells will reduce the burden of the neurodegenerative disease.

## 9. Multiple Targets of Polyphenols against AD

**Figure 2:**
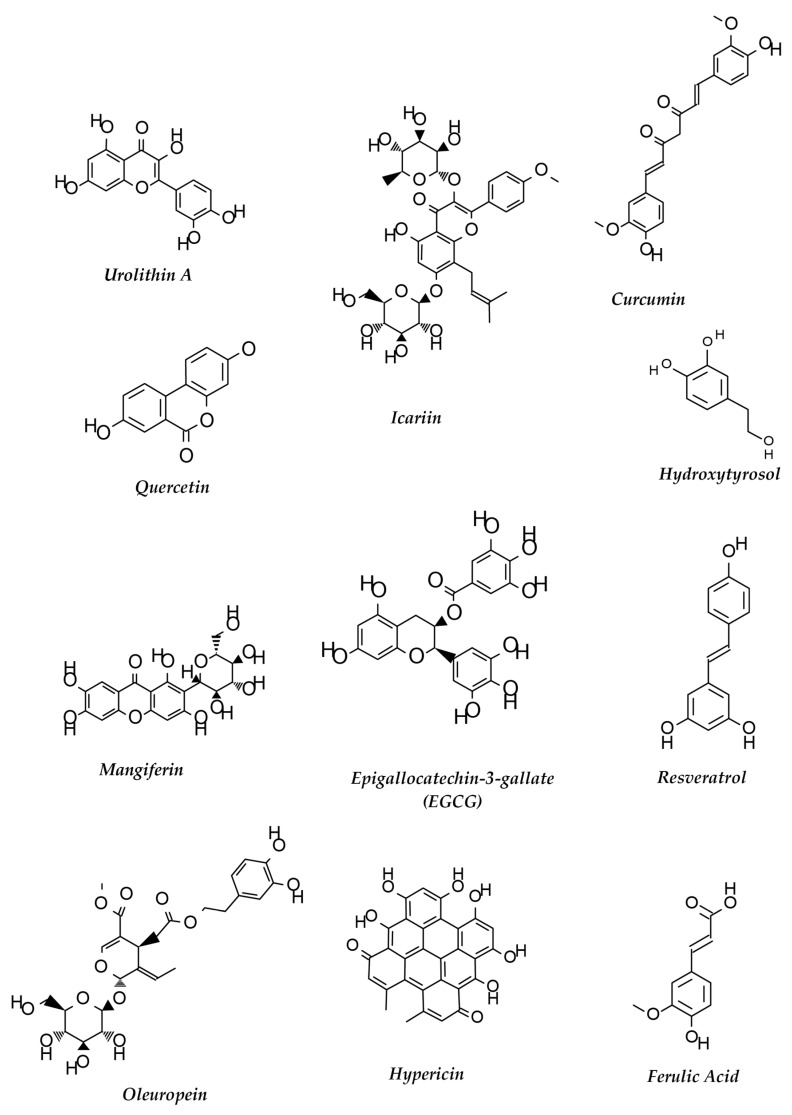
Structures of some polyphenols that show neuroprotective functions against AD.

Polyphenols are a class of compounds that are commonly found in many plants. Four major classes of polyphenols including flavonoids, stilbenes, phenolics and lignans are highly regarded as potential therapeutics for neurodegeneration, cardiovascular diseases, cancer and obesity. Many more polyphenolic compounds are yet to be studied for their potency in AD and other neurodegenerative diseases. Polyphenols are classified according to their structure (reviewed in [265]). Structures of some important polyphenols that are described in the text are depicted in Figure 2.

Polyphenolic compounds abound in mushrooms and are one of their main antioxidants. They are mainly phenolic acids which can be divided into groups of either hydroxybenzoic acids and hydroxycinnamic acids derived from the non-phenolic molecules benzoic and cinnamic acid, respectively [266]. The most common benzoic acid derivatives present in mushrooms were reported as *p*-hydroxybenzoic, protocatechuic, gallic, gentisic, homogentisic, vanillic, 5-sulfosalicylic, syringic, ellagic and veratric acids as well as vanillin. Meanwhile, cinnamic acid derivatives mainly found in mushrooms were *p*-coumaric, *o*-coumaric, caffeic, ferulic, sinapic, 3-*o*-caffeoylquinic, 4-*o*-caffeoylquinic, 5-*o*-caffeoylquinic and tannic acids [266].

It is known that only plants synthesize flavonoids, while animals and fungi are not capable of it. However, accumulating studies indicate the presence of flavonoids in different edible mushrooms [267]. The presence of flavonoids in mushrooms could arise from absorption from the substrates where they grow or from neighboring plants by establishing symbiotic interactions via formation of mycorrhizae [268].

### 9.1. Polyphenols as Antioxidants

Naturally occurring polyphenols provide protection against neurodegeneration through their role as antioxidants [269]. Dietary polyphenols have direct ROS scavenging activity [270]. Several polyphenolic antioxidants identified in common edible mushrooms include protocatechuic acid, *p*-coumaric, and ellagic acid as well as gallic acid, pyrogallol, homogentisic acid, 5-sulfosalicylic acid, chlorogenic acid, caffeic acid, ferulic acid and quercetin [271,272]. Most of these polyphenols donate electrons to the free radicals thus neutralizing them, which ultimately reduces the levels of ROS within the cells. Polyphenols activate Nuclear factor erythroid 2-related factor 2 (Nrf2), a basic leucine zipper transcription factor. Nrf2 normally is complexed with Kelch-like ECH-associated protein 1 (Keap1) in the cellular environment inhibiting Nrf2′s nuclear translocation. Furthermore, Keap1 also facilitates ubiquitination and proteasomal degradation of Nrf2 [273]. The separation of Nrf2 from Keap1 leads to activation and nuclear translocation of Nrf2, where it complexes with musculoaponeurotic fibrosarcoma (Maf) proteins. This heteromeric Nrf2-Maf complex then binds with antioxidant response element (ARE) sequences located upstream to the phase II detoxifying genes upregulating their expression. Phase II antioxidant genes encode proteins, such as heme oxygenase 1, γ-glutamyl cysteine synthetase, peroxiredoxins, glutathione reductases, thioredoxin reductase, drug metabolizing and detoxification enzymes NAD(P)H quinone dehydrogenase 1, glutathione-*S*-transferase, uridine diphosphate-glucuronosyltransferase and regulators, transketolase, PPARγ-coactivator 1 β (PGC1-β), etc [274]. These proteins act in the cell as antioxidant proteins, having a major role in restoration of the redox imbalance and cellular signaling [275,276]. Additionally, polyphenols also elucidate their antioxidant property through inhibition of NADPH oxidase (NOX) activities [277]. NOX proteins are transmembrane proteins that signal the immune modulators through ROS generation [278]. Lower levels of ROS may be important for cellular signaling, however, at higher levels they can cause damage to the neuronal cells. These proteins, found to be involved in increasing Aβ-induced oxidative stress, could be potential therapeutic targets for AD [279].

Oxidative damage is more prominent when the damage is coupled with mitochondrial dysfunction. Enzymes such as monoamine oxidases (e.g., MaoB) increase the cellular stress by producing hydrogen peroxide [280]. In brains, monoamine oxidase activity of substrate neurotransmitters causes mitochondrial damage, while dietary polyphenols have been found to inhibit MaoB, thus decreasing the ROS generation and mitochondrial dysfunction [36]. Additionally, polyphenols also aid in regeneration of mitochondria in the cells through activation of the master regulator SIRT1 [281]. SIRT1 is a NAD^+^-dependent histone deacetylase enzyme that has multiple targets for deacetylation. SIRT1′s involvement in reducing oxidative stress comes from deacetylation of its substrate PGC-1α, which activates nuclear respiratory factors (Nrf1 and Nrf2) and peroxisome proliferator-activated receptor (PPARα) [282]. Further downstream, these molecules enhance the expression of transcription factor A, mitochondrial (TFAM) that initiates the transcription and replication of mitochondrial DNA ultimately causing the regeneration of mitochondria [282]. The activation AMPK, either directly or indirectly (through SIRT1 activation) activates PGC-1α, thus helping in mitochondrial biogenesis.

**Figure 3:**
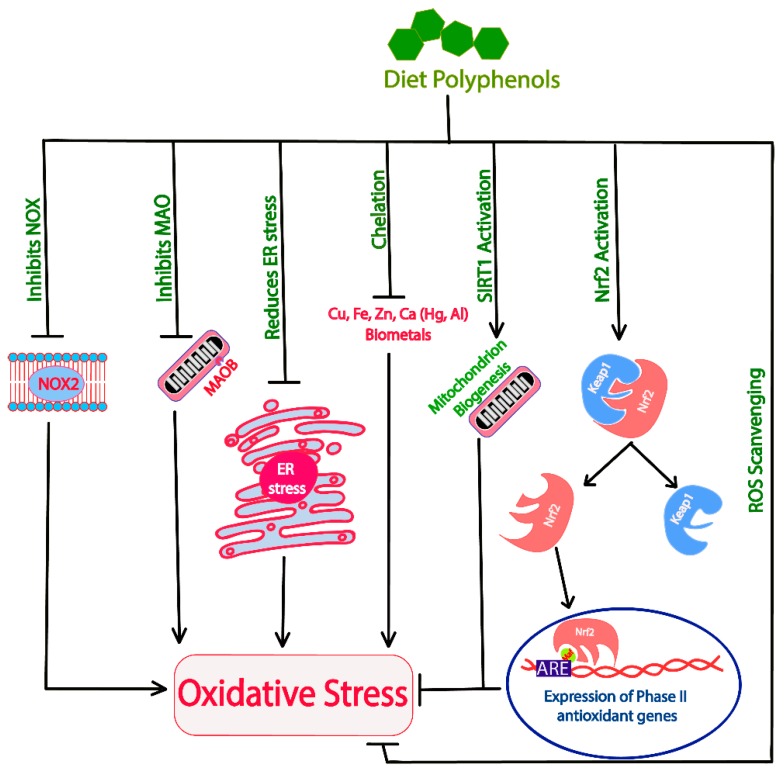
Schematic representation for showing molecular mechanisms by which polyphenols acts as antioxidants (adapted from [36,270,273,274,277,281,284]).

Biometals such as iron and copper are the major contributors of ROS formation in defunct mitochondria [283]. Quercetin, baicalein, curcumin, etc., are found to provide a protective antioxidant property also through biometal chelation [4,284,285]. Furthermore, alterations in biometal distribution in the neuronal cells is also an important hallmark of AD. The mechanism through which polyphenols act as antioxidants in the cellular environment is schematically presented in Figure 3. Antioxidants can also act as pro-oxidants in certain sub-optimal concentrations and cause oxidative damage to the cells. Thus, their optimum concentration needs to be considered prior to their application.

### 9.2. Modulation of Protein Homeostasis and Longevity with Polyphenols

Dietary polyphenols modulate the protein quality control mechanisms increasing the cellular efficiency to clear misfolded proteins. Apart from induction of autophagic clearance, the UPR and ubiquitin proteasome system are also modulated by dietary polyphenols [286,287,288]. The ability of polyphenols to activate lysosomal biogenesis and increase longevity make them an important class of neuroprotective compounds [5,34,37]. In addition, some of the polyphenols like EGCG and curcuminoids reduced the lipofuscin granules in cells, which normally are impossible to degrade or exocytose from the cell [15,289]. Reduction of lipofuscin in the cell can contribute to the restoration of the protein homeostasis by reducing the damage to autophagosomes and proteasomes.

**Figure 4:**
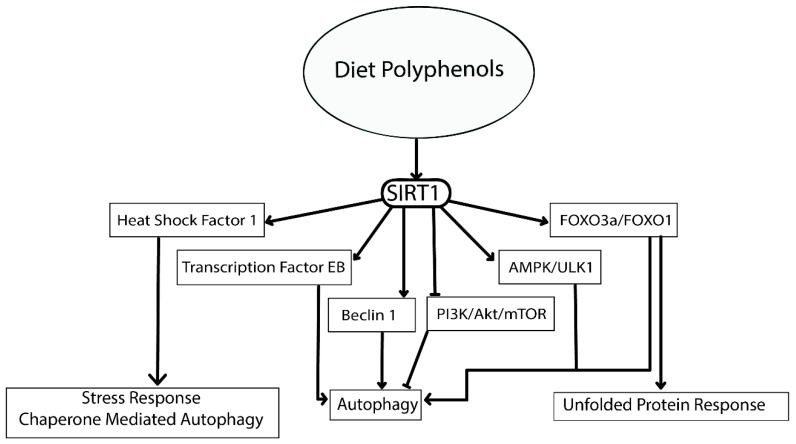
SIRT1 activation by polyphenols and its effect in protein degradation pathways in the intracellular environment (adapted from [37,290,291,292,293,296]).

Most of the polyphenolic compounds act through upregulation of the expression of the master regulator SIRT1 [290]. The SIRT1 protein has been found to have multiple targets that play a vital role in regulating major cellular processes (refer to Figure 4) [290]. The activation of AMPK/Unc-51 like autophagy activating kinase 1 (ULK1), transcription factor EB (TFEB), Fork head box O transcription factors (FOXO), deacetylation of p53 and inhibition of PI3K/Akt/mTOR, NFkB, MAPK and the c-Jun N-terminal kinases (c-JNK) pathway are important cellular processes that will induce autophagy through SIRT1 [291,292,293]. Most of these molecular targets are deacetylation substrates of SIRT1. Activation of transcription factors like TFEB reinforces the cellular autophagy by activating lysosomal biogenesis. TFEB itself is another master regulator for the coordinated lysosomal expression and regulation (CLEAR) network. The CLEAR network has important roles in various cellular processes. Energy metabolism, DNA metabolism, steroid biosynthesis, protein clearance, haemoglobin degradation, antigen presentation, phagocytosis and signal transduction are important events regulated by TFEB [294,295].

**Figure 5:**
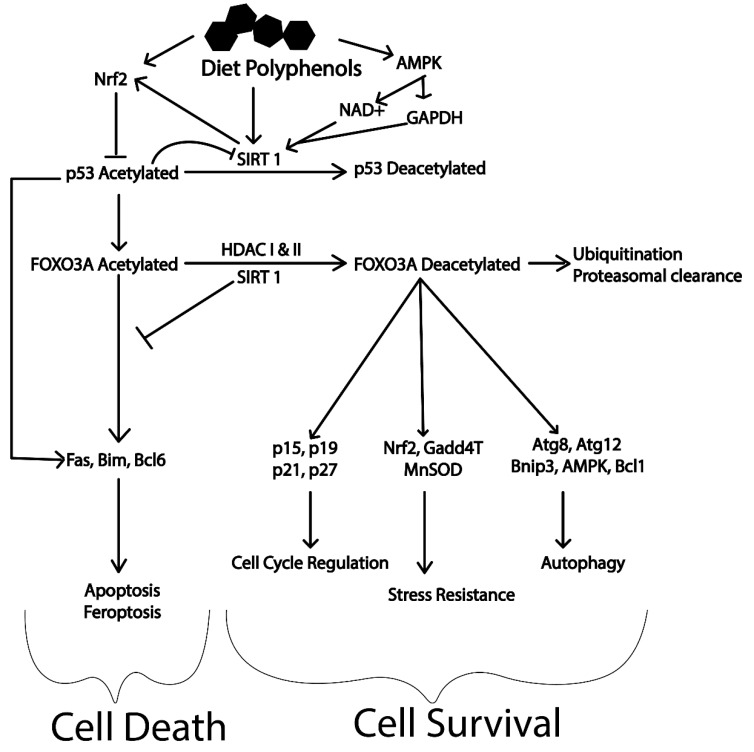
Modulation of longevity by the action of polyphenols through SIRT1 activation (adapted from [288,291,292,295,296,297,298]).

Similarly, SIRT1 has a significant role in determining cellular fate via Fork head transcription factors (FOXO1 and FOXO3). The deacetylated form of these transcription factors are major contributors of autophagy activation, cell cycle arrest, stress resistance (expression of manganese superoxide dismutase) and immune modulation. Reduction in the levels of FOXO by ubiquitination and proteasomal degradation with the help of SIRT1 reduces the levels of acetylated forms. Reduction in acetylated FOXO’s suppresses cell death caused by apoptosis driving cells towards survival and increasing longevity (refer to Figure 5) [292,296]. This is of particular interest for neurodegenerative diseases, where survival of neuronal cell after damage is crucial. It has been illustrated that polyphenols activate these master regulators of longevity (Nrf2, SIRT1 and AMPK) providing unprecedented protection against various disease [276,297,298]. However, limited bioavailability of these dietary polyphenols in human has limited their application. Polyphenols such as hydroxytyrosol, oleuropein aglycone, curcumin, resveratrol, rotenone, rutin, myricetin, urolithin A, epigallocatechin 3-gallate (EGCG), ferulic acid, genipin, etc. have been reported to induce autophagy. The olive oil polyphenol, hydroxytyrosol activates AMPK pathway and is reported to reduce Aβ levels in mouse models of AD [28,299]. Similarly, oleuropein aglycone has been reported to activate SIRT1/AMPK/mTOR and TFEB mediated autophagy [300,301].

Curcumin, one of the most studied polyphenols, has multifactorial benefits in balancing the protein homeostasis by activation of AMPK/ULK1 and inhibition of PI3K/Akt/mTOR through activation of SIRT1 [38]. EGCG, a catechin family polyphenol, inhibits the suppressors (Bcl2 and Bcl-XL) of Beclin1. However, the activity of this polyphenol is also dependent on the concentration of the compound. A higher concentration of EGCG inhibits autophagy and induces apoptosis, whereas, lower concentrations induce autophagy that also degrade lipid droplets through a Ca^2+^/CAMKKB/AMPK dependent mechanism. Thus, the concentration of polyphenols is a crucial factor before considering it as a therapeutic option. EGCG has also been reported to reduce the catalytic activity of 19S and 20S proteasomal proteins, deactivate NFkB pathway and enhance p53 tumour suppressor protein expression [302]. An important feature of EGCG also includes its ability to inhibit lipofuscin formation, which otherwise impairs autophagy and the proteasome during ageing [15].

Resveratrol is another important polyphenol frequently studied for its beneficial effect in increasing longevity and balances cellular protein homeostasis. The activation of SIRT1/AMPK and extracellular signal-regulated kinases (ERK1/2) is the molecular mechanism by which this polyphenol was found to be neuroprotective [9,303,304]. The metabolite of ellagitannin, urolithin A, extracted from pomegranate has been reported to activate autophagy through SIRT1 activation [305]. Furthermore, the natural compound was also found to increase mitophagy and longevity in a *Caenorhabditis elegans* (*C. elegans*) model that has provided insight on human neurodegeneration [49]. Quercetin has shown multiple benefits in human health by enhancing autophagy through SIRT1activation, inhibiting proteasomal degradation (inhibition of all the catalytic subunits), reducing proliferation and activating apoptosis [306]. Apart from autophagy inducers, hesperitin and hesperidin have also been reported to have negative effects on Aβ-induced autophagy and glucose metabolism impairment [307,308].

### 9.3. Polyphenols and Cellular Lipid Balance

Polyphenols are also considered as potential therapeutic agents against obesity and other life-threatening conditions [309,310,311]. This property of polyphenols is associated with the activation of AMPK, which targets lipid metabolism as well [312]. Activation of AMPK decreases the activity of acetyl CoA carboxylase, HMG-CoA reductase and diacylglycerol acyl transferase, and thus avoids hepatic accumulation of lipids [313,314]. These actions of AMPK reduce the levels of free fatty acids as well as the complex lipids. Polyphenols are also found to inhibit the adipogenesis by inhibiting proteins like PPARγ [315,316]. Additionally, as explained in previous sections, polyphenols increase autophagic clearance. Induction of autophagy is not only limited to restoring the protein balance but is also associated with the degradation of lipids to meet the energy demands of the cells. Thus, polyphenols can also reduce lipid accumulation in the intracellular environment [260]. AD is also termed as Type III diabetes due to its similarity with diabetes. High levels of cholesterol have been found to be associated with AD brains [317]. Lowering the levels of cholesterol has been an important approach for the treatment of AD, despite limited success. Furthermore, studies support increased activity of γ-secretase and β-secretase with higher levels of lipids in the membrane environment that could contribute to increased Aβ levels in the brain [210]. Considering these facts, polyphenols are hypothesized to have their neuroprotective action in part through the restoration of lipid homeostasis.

### 9.4. Anti-inflammatory Activity of Polyphenols

ROS act as signaling molecules for induction and release of pro-inflammatory mediators including NFκB and cytokines. NFκB exists in an inactivated form bound to an inhibitor referred to as p65/p50 dimer in normal conditions [318]. When this complex gets activated by increased ROS, the p65/p50 dimer translocates to the nucleus upregulating expression of the inflammatory markers [319]. The expression of these inflammatory mediators inside the cells triggers the downstream process of inflammation. Deacetylation of NFκB through the action of SIRT1 at specific amino acid residues renders it inactivated and reduces the inflammatory response by reducing the expression of downstream genes [318]. Since polyphenols are antioxidants capable of lowering the ROS in the cells, they can downregulate the expression of proinflammatory mediators [320]. However, the highest anti-inflammatory activity of polyphenols is attributed to their ability to activate the master regulator SIRT1 [321]. Many polyphenols have been reported to have an anti-inflammatory effect which could provide the basis for protection against diseases with chronic neuroinflammation/inflammation.

### 9.5. Polyphenols as Anti-amyloid Agents

Oleuropein, an olive polyphenol, is found to increase α-secretase activity. Thus, it prevents cells from producing Aβ: instead such activity results in the formation of the Aα peptide [322]. Formation of Aα instead of Aβ is anti-amyloidogenic, which may be helpful in reducing the Aβ-associated toxicity. Some polyphenols (such as rutin) reduce the β-secretase activity [6]. Similarly, other polyphenols disaggregate the amyloid aggregates *in vitro* [6,323]. Furthermore, the ability of polyphenols to lower the cholesterol levels in cells also favors the reduced activity of β-secretase and γ-secretase [6,317]. Apart from the anti-amyloid functions, polyphenols also possess the ability to inhibit tau aggregation [324].

Through characterization of the cell-free extracts of different bacteria, fungi and yeast, Lee *et al*. (2007) identified the BACE1 inhibitory effects of different mushrooms [325]. Mushroom species having anti-BACE1 effects were *Flammulina velutipes*, *Pleurotus ostreatus*, *Grifola frondosa*, *Dictyophora echinovolvata*, *Fomitella fraxinea* and *Inonotus obliquus*. Hispidin, a polyphenolic compound found in abundance in the mushroom *Phellinus linteus* inhibits BACE1 non-competitively and scavenges free radicals [326]. BACE1’s inhibitory effect of *Auricularia polytricha* has also been indicated to be hispidine mediated [327].

### 9.6. Polyphenols in Cognition and Synapsis

Polyphenolic compounds like α-isocubebenol, tacrine and their derivatives, 2′,4′-dihydroxy-6′methoxy-3′,5′-dimethyl-dihydrochalcone, tetrahydropyranodiquinolin-8-amines, quercetin and tiliroside have been shown to have neuroprotective properties attributed to their inhibiting activity against acetylcholine esterase [328,329,330,331]. In addition, some other polyphenols, including genistein, luteolin-7-O-rutinoside and silibinin, are reported to have a moderate effect on the butyrylcholine esterase [330]. Among the polyphenols, flavonoids are an important class of polyphenols that have anti-choline esterase activity [167]. Flavonoids extracted from *Ginkgo biloba* have been reported to have inhibitory effects against acetyl choline esterase [168]. Molecular docking experiments revealed the mechanism of action of quercetin was through strong hydrogen bond formation with certain amino acids of AChE, thus leading to competitive inhibition of AChE. Similarly, macluraxanthone exhibited non-competitive type interference with the activity of acetyl choline esterase [167]. The combination of numerous hydrogen bonds with several amino acids and hydrophobic interaction may be responsible for how these polyphenols inhibit acetylcholine esterase activity [332].

Polyphenols exert neuroprotective effects in experimental systems but there is a need to translate this in guidelines for neuroprotection of aging populations. For translation of animal studies to human trials, dose accuracy plays a critical role. For example, consider resveratrol levels in Table 1: an effective dose in mice is 60 mg/kg/d by oral administration. In humans this translates to ~290 mg for a 60 kg person per day [333]. Such levels are rarely reached. In the case of resveratrol, the suggested daily intake is 200 mg/day and this is unlikely to be a protective level. In addition, alterations in polyphenol administration routes may reduce the amount of polyphenol to be used on daily basis, signifying the benefits of alternative administration strategy. However, long term uptake of the polyphenol could still have beneficial effects in lower doses. On the other hand, some nutraceutical products may contain the polyphenol at more than the optimal amount, which could have negative effects in brain health [334]. This bimodal activity of polyphenols should be highly considered before translating the beneficial effects of the polyphenols for human use.

## 10. Future Directions

In order to reap the full benefits of polyphenols as therapies in AD, some limitations should be considered—especially in regard to safety, pharmacokinetics, bioavailability, delivery system, administration route, dose efficiency and clinical status (reviewed in [334]). In terms of safety, polyphenols were generally regarded as safe and well-tolerated in animals as well as humans with no notable side effects even for high and repeated dosages [335]. If any, side effects are usually mild, tolerated, and transient: for instance, minor headaches, dizziness, gastrointestinal problems, and skin rashes. Another important point to contemplate is the possible interaction of clinically-prescribed drugs with polyphenols, as polyphenols are currently viewed as nothing more than a supplement, and far from being a substitute for prescription drugs. For example, flavonoids in grapefruit juice demonstrated potent inhibition of the cytochrome P450 (CYP) protein family, critical for drug metabolism. The abrupt inhibition of CYP may potentially lead to excessive buildup of drugs increasing toxicity [336,337,338]. Regardless, polyphenols taken exclusively were harmless either in short, medium, or long-term supplementation in humans [339,340,341,342,343,344,345], which certainly encourages their application. Despite countless attempts proving the AD-ameliorating efficacy of polyphenols in a wide range of *in vitro*, *in vivo*, and epidemiological studies, the translation into human trials is indeed difficult, and failure was common in the early stages of most clinical trials [346]. However, their efficacy has improved over time with further modification of multiple factors, including effective dosage and period of administration. As a result, resveratrol [347,348] and *Ginkgo biloba* (flavone glycosides and terpene lactones) [343,345,349] showed promising results in the initial phases, while EGCG stands out by reaching phase III of clinical trials [350]. There is no doubt about the benefits and potential of polyphenols in the management of AD, but a poor understanding of pharmacokinetics and pharmacodynamics has restricted their applicability. In many instances, their bioavailability in the CNS was limited due to low absorption in the gastrointestinal tract, rapid metabolism, systemic elimination, and impermeability across the BBB [351,352,353]. Processing and first-pass metabolism of these dietary polyphenols, which occurs at different levels, including the stomach, small intestine, large intestine, circulatory system and liver, may cause significant changes in polyphenol structure, quantity and biological activity [354,355]. Furthermore, the gut microbiome also takes part in metabolizing these bioactive compounds [355,356]. Studies suggest only 5-10% of the dietary polyphenols are absorbed, leaving much room for improvements to increase the bioavailability of these potential therapeutics. Even more critical in neurodegenerative disorders is the requirement for these polyphenols to cross the BBB from the bloodstream to the brain tissue to reach their target, which depends on their lipophilicity [357,358]. Hence, future research should be focused on optimizing the bioavailability of these compounds in the human body, particularly in brain tissues, to have enhanced effects. Recent studies involving the encapsulation of these bioactive compounds into stable nanoparticles and microparticles could be significant [359]. The possibilities of administering these compounds through a different route into the human body should be considered: for instance, intranasal or intravenous administration to avoid inactivation during the first-pass metabolism and gut microflora intervention. Improvement in targeted delivery through engineering particles in such a way that their bioavailability is increased would be the basis for further research. Considering these facts improvements made to enhance the bioavailability of curcumin [360,361] and resveratrol [362] were successful to some extent, which provides a roadmap for future studies.

## Figures and Tables

**Table 1:** Neuroprotective roles of some polyphenols for AD.

Polyphenol	Analytical System	EPC^a^/ROA^b^	Effects of Polyphenols at Cellular Level	Effects in Relation to AD	Reference
Quercetin	*In vitro*	NA	mTORC inhibitor	Induces autophagy, anti-amyloidogenic, inhibits proteasomal degradation, antioxidant, restores biometal distribution, antiproliferative and enhances neuronal synapsis	[4,5,6,7,8]
	ARPE 19 cells	2 μM	TFEB activation		
	APPswe cells	10 μM	Inhibits Aβ fibril formation		
	Rat neonatal cardiomyocytes	5 μM	Inhibits all the catalytic subunits of proteasome		
	*In vitro*	NA	Chelates iron		
	*In vitro*	NA	Reduces ROS and RNS		
	*In silico* and *in vitro*	NA	Inhibits acetyl choline esterase		
Resveratrol	Tg6799 mice	60 mg/kg/d for 60 d/oral administration	Reduces amyloid plaque formation	Induces autophagy, increases lysosomal biogenesis, restores lipid homeostasis, increases stress resistance, regulates cell cycle, antiproliferative, anti-apoptotic, increases longevity and anti-inflammatory	[9,10,11,12,13,14]
	Primary neuronal culture	30 μM	SIRT1 activation and NFκB inhibition		
	Obese healthy men clinical trial	150 mg/d for 30 d/oral administration	TFEB activation		
	Human aortic endothelial cells	50 μM	AMPK mediated LC3II activation		
	Human aortic endothelial cells	10 μM	Decreases ROS and RNS, increases SOD		
	LNCaP cells	20 μM	p53 regulation, PI3K/Akt/mTOR inhibition, induces FOXO transcriptional activity including cell cycle regulation and stress resistance		
Epigallocatechin gallate (EGCG)	Human bladder cancer cell line T24	20 μg/ml	Inhibits Beclin1 suppressors and PI3K/Akt/mTOR	Induces autophagy, restores lipid homeostasis, anti-amyloidogenic, increases antioxidant capacity, restores impaired autophagosomes and biometal distribution, increases cell survival	[15,16,17,18,19]
	Bovine aortic endothelial cells	10 μM	Increases LC3II formation and activates AMPK/ULK1		
	HepG2 cells	40 μM	Degrades lipid droplets through Ca^2+^/CAMKKB AMPK dependent mechanism		
	*In vitro*	NA	Chelates zinc and copper		
	PC12 cells (rat pheochromocytoma	100 μg/mL	Interacts with Aβ40 and changes its conformation, inhibits lipofuscin formation		
Anthocyanin	Sprague–Dawley rats	100 mg/kg/d for 28 d/oral administration	Restores calcium homeostasis and activates Nrf2 subsequently activating phase II detoxifying genes	Activates autophagy, increases expression of anti-oxidant genes, reduces ROS and increases cell survival	[20,21,22,23]
	HT22 cells and primary cultures of hippocampal neurons	0.1 mg/mL	Induces AMPK		
	*In vitro*	0.005 mg/mL	ROS scavenging		
	HCC cell lines PLC/PRF/5 and HepG2 cells	0.2 mg/mL	Increase expression of Beclin1, LC3 II		
Kaempferol	SK-HEP-1 human hepatic cancer cell	75 μM	Increases the levels of p-AMPK, LC3-II, Atg 5, Atg 7, Atg 12 and beclin 1, inhibits PI3K/Akt/mTOR	Reduces mitochondrial dysfunction, anti-proliferative, increases autophagy, increases unfolded protein response, reduces APOE4 fragmentation and associated toxicity	[24,25,26,27]
	BALB/c nude mice	150 mg/kg/d for 31 d/intraperitoneal injection	Activates DNMT methyltransferase ubiquitination		
	SCC-4, human tongue squamous cell carcinoma cell	50 µM	Activates IRE1-JNK-CHOP signaling, downregulates ERK1/2 signaling which reduces MMP2		
Hydroxytyrosol	Male db/db (C57BL/6J) mice	10 mg/kg/d for 8 weeks/oral administration	Activates Nrf2 and SIRT1/AMPK/PGC-1, reduces protein oxidation, increases NMDAR1 and NGF mRNA expression	Enhances autophagy, increases stress resistance and longevity, antioxidant, anti-inflammatory, restores lipid homeostasis and improves cognition	[28,29,30,31,32,33]
	VECs cells	50 μM	Activates AMPK/FOXO3a		
	VECs cells	10 μM	Reduces ROS		
	VAFs from Sprague–Dawley rats	25 μM	Increases LC3II/LC3I, Bcl1 and SIRT1 expression		
	HepG2 and Huh7 cells	100 μM	Inhibits PI3K/Akt/mTOR, expression of IL1β & IL6, and NFκB DNA binding		
	Rat hepatocytes	25 μM	Inhibits Acetyl CoA carboxylase, HMG CoA reductase, diacylglycerol acyl transferase		
Oleuropein aglycone	Rat ventricular myocyte	100 μM	Increases Bcl1 and LC3II expression, TFEB nuclear localization, LAMP1 and p62 expression	Induces autophagy, increases lysosomal biogenesis and reduces oxidative damage	[33,34,35]
	Human SH-SY5Y neuroblastoma cells and rat RIN5F insulinoma cells	50 μM	Inhibits MAOA, induces AMPK/ULK1, inhibits mTOR		
	Rat hepatocytes	25 μM	Inhibits acetyl CoA carboxylase, HMG CoA reductase and diacylglycerol acyl transferase		
Curcumin	Male Sprague–Dawley rats	15 mg/kg/d for 4 weeks/subcutaneous injection	Activates AMPK and regulates lipid metabolism	Induces autophagy, restores lipid homeostasis, antioxidant, anti-amyloidogenic, anti-inflammatory, anti-apoptotic antiproliferative, increases lysosomal biogenesis and longevity	[36,37,38,39,40,41,42,43,44,45]
	Adult male Wistar rats	30 mg/kg for 30 d/oral administration	Activates Nrf2, inhibits NFκB and mTOR		
	Adult Swiss male albino mice	80 mg/kg/d for 7 d/intraperitoneal injection	Inhibits MaoB and reduces ROS		
	APPswe Tg2576 transgenic mice (chronic 500 ppm curcumin diet)	Blood curcumin level ~2 μM for 1 h/injection in right carotid artery	Inhibits formation of Aβ, oligomers, fibrils and plaques		
	Tsc2^+/+^, Tsc2^−/−^ MEFs and HCT116 cells	10 μM	Activates TFEB, increases levels of LC3 and inhibits pAkt		
	Sprague–Dawley rats’ primary cortical neurons	10 μM	Upregulates SIRT1 and inhibits Bax		
	APP/PS1 double transgenic mice	160 ppm for 6 months/oral administration	Inhibits PI3K/Akt/mTOR signaling, increases LC3I/II and Beclin1 expression		
Myricetin	HepG2 Cells	50 μM	Inhibits mTOR and increases LC3II expression	Induces autophagy, antiproliferative, increases stress resistance, longevity, antioxidant capacity and mitochondrial regeneration	[46,47,48]
	Adipocytes differentiated from C3H10T1/2 cells	10 μM	Activates SIRT1/SIRT3/SIRT5		
	Male ICR mice	50 mg/kg/d for 21 d/oral administration	Increases mitochondrial mass and increases PGC1α, SIRT1, TFAM, Nrf1 & FOXO1		
Urolithin A	C2C12 myoblasts	50 μM	Induces mitophagy, increases LC3I/LC3II and activates AMPK signaling	Increases mitophagy, and autophagy, antioxidant, increases lysosomal biogenesis, anti-inflammatory, anti-amyloidogenic, improves cognition and longevity	[49,50]
	Female APP/PS1 transgenic mice B6C3-Tg (APPswe, PS1dE9) 85Dbo/J and age-matched wild type mice	300 mg/kg/d for 14 d/oral administration	Activates AMPK, decreases NFκB/MAPK/BACE1 activities and APP levels		
Ferulic Acid	HeLa cells and mouse primary hepatocytes	1 mM	Increases LC3 II and inhibits mTOR	Anti-apoptotic, anti-amyloidogenic, antioxidant, anti-inflammatory and induces autophagy	[51,52,53,54]
	*In vitro*	NA	Inhibits Aβ aggregation and reduces ROS		
	(APP)swe/presenilin 1(PS1)dE9 (APP/PS1) mouse model	5.3 mg/kg/d for 6 months/oral administration	Reduces amyloid deposition and interleukin-1 beta (IL-1β) levels		
Acacetin	*Drosophila melanogaster*	100 μM	Inhibits BACE1	Anti-amyloidogenic, antioxidant, anti-inflammatory and induces autophagy	[55,56,57,58]
	C57BL/6J mice	∼10 mg/kg/d for 14 d/oral administration (gavage)	Inhibits MAPK and PI3K/Akt pathways		
	ICR mice	100 mg/kg for 7 h/intraperitoneal injection	Increases LC3II, Atg5 and Atg7 expression, modulates TNF-α/IL-6 expression and suppresses TLR4 signaling		
Baicalein	SH-SY5Y human neuroblastoma cells	12.5 μM	Increases ROS scavenging and activates Nrf2	Anti-amyloidogenic, anti-apoptotic, antioxidant, anti-inflammatory, inhibits excitotoxicity, stimulates neurogenesis and neuronal differentiation	[59,60,61,62,63,64,65]
	*In vitro*	NA	Chelates iron		
	CHO/APPwt cells	5 μM	Induces α-secretase and inhibits Aβ formation		
	*In vitro*	30 μM	Dissociates amyloid aggregates, Aβ oligomerization and fibrillation		
	HeLa cells	100 μM	Inhibits NFκB activation		
	C57BL/6J APP/PS1 mice	80 mg/kg/d for 60 d/oral administration (drinking water)	Inhibits GSK3β mediated tau phosphorylation		
	Sprague-Dawley male rats	20 mg/kg 30 min before and 2/4 h after onset of ischemia/intraperitoneal injection	Induces Bcl-2/Bcl-xL associated phosphorylation		
Icariin	Primary cortical neurons prepared from E16-17 mouse embryos	1.2 μM	Activates SIRT1	Antioxidant, anti-amyloidogenic, reduces ER stress, increases synapsis and neuronal plasticity, inhibits tau hyperphosphorylation, increases cell viability, antiapoptotic and anti-inflammatory	[66,67,68,69,70,71,72,73]
	Wistar rats	60 mg/kg/d for 3 months/oral administration	Increases SOD activity		
	Tg2576 mouse model	60 mg/kg/d for 3 months/oral administration	Reduces expression of BACE1 and APP		
	Sprague-Dawley rats	120 mg/kg/d for 28 d/oral administration	Induces PSD95, BDNF, pTrkB, pAkt, and pCREB expression		
	SH-SY5Y cells	1 μM	Inhibits GSK3β activation		
	PC12 cells	10 μM	Inhibits JNK/p38, MAPK and p53 activity		
	HT29 and HCT116	20 μM	Inhibits NFκB signaling		
Nobiletin	Male 3XTg-AD mice	30 mg/kg/d for 3 months/intraperitoneal injection	Reduces Aβ levels and plaque formation in brain	Anti-amyloidogenic, increases stress resistance, neuronal synapsis and plasticity, antioxidant and anti-inflammatory	[74,75,76,77]
	Male Sprague-Dawley rats	25 mg/kg/d for 3d/intraperitoneal injection	Increases activity of Akt, CREB, BDNF and Bcl2, increases Nrf2, HO-1, SOD1 and GSH expression, reduces NFκB, MMP-9 and MDA expression		
Genistein	*In silico* and *in vitro*	NA	Inhibits chymotrypsin-like activity of proteasomes	Antioxidant, increases degradation of Aβ, increases apoptosis, enhances autophagy and inhibits proteasomal protein degradation	[78,79,80,81]
	LNCaP cells	100 μM	Increases Kip1 and reduces IκBα/Bax		
	Human dermal fibroblasts (HDFa)	30 μM	Increases TFEB expression		
	Human mammary gland tumor cells (MCF-7)	0.5 μM	Enhances antioxidant gene expression		
Luteolin	HT-29 cells	50 μM	Reduces ROS, NFκB signaling, Cox2 expression, blocks JAK/STAT signaling	Anti-inflammatory, antioxidant, modulates autophagy and apoptosis, increases survival	[82,83,84,85,86]
	Male Sprague-Dawley rat myocytes	8 μM	Downregulates Bax expression, upregulates PI3k/Akt signaling and Bcl-2 expression		
	Human HCC cell line SMMC-772	100 μM	Increases expression of LC3B-II, Bcl1 and caspase 8		
Mangiferin	Swiss albino male rats	15 mg/kg/d for 14 d/intraperitoneal injection	Increases ROS scavenging, activates Nrf2, inhibits NFκB signaling, increases GSH levels, decreases lipid peroxidation	Antioxidant, anti-apoptotic, chelates metals, increases stress resistance, autophagy, longevity, neuronal synapsis and plasticity	[87,88,89,90,91]
	*In vitro*	NA	Rescues mitochondrial respiration, chelates iron		
	Male Swiss albino mice	40 mg/kg/d for 21 d/oral administration	Reduces lipid peroxides and ROS/RNS induced by aluminum and restores regulation of BDNF and NGF		
	Human astroglioma U87MG, U373MG and CRT-MG cells	100 μM	Inhibits PI3K/Akt signaling, MAPK pathway, MMP9 gene expression		

Footnotes: NA, not applicable; ^a^ EPC, minimum concentration of the polyphenols that have significant neuroprotective effect; ^b^ ROA, route of administration of polyphenols in *in vivo* models.

## Abbreviations

AD	Alzheimer’s Disease
Aβ42	β-amyloid of 42 amino acids
Aα	Amyloid α
NGF	Nerve Growth Factor
FDA	Food and Drug Administration
NMDAR	N-Methyl-D-Aspartic Receptor
APP	Amyloid Precursor Protein
BACE	β-Secretase
NFT	Neurofibrillary Tangle
NFκB	Nuclear factor kappa B
MAPK	Mitogen-Activated Protein Kinase
GSK3β	Glycogen Synthase Kinase - 3β
CDK	Cyclin dependent kinase
APOE	Apolipoprotein E
ROS	Reactive Oxygen Species
RNS	Reactive Nitrogen Species
GABA	γ-amino butyric acid
cGMP	Cyclic guanosine monophosphate
cAMP	Cyclic adenosine monophosphate
COX	Cyclooxygenase
PPARγ	Peroxisome proliferator-activated receptor γ
FAAH	Fatty acid amide hydrolase
MAGL	Mono acyl glycerol lipase
BDNF	Brain-derived neurotrophic factor
NT	Neurotrophin
Trk	Tropomyosin receptor kinase
PI3K	Phosphatidylinositol-3-kinase
Akt	Protein kinase B
BBB	Blood Brain Barrier
ECB	Encapsulated Cell Bio-delivery
AChE	Acetylcholine esterase
MAO	Monoamine oxidase
UPR	Unfolded protein response
IRE	Inositol response element
ATF	Activating transcription factor
PERK	Protein kinase RNA-like endoplasmic reticulum kinase
LAMP	Lysosome associated molecular pattern
ATP	Adenosine triphosphate
AMPK	Adenosine monophosphate kinase
mTOR	Mechanistic Target of Rapamycin
NADPH	Dihydronicotinamide-adenine dinucleotide phosphate
NOX	NADPH oxidase
TFEB	Transcription factor EB
SIRT1	Sirtuin 1
FOXO	Fork head box like protein O
Nrf	Nuclear factor erythroid-2 related factor
Keap	Kelch-like ECH-associated protein 1
Maf	Masculoaponeurotic fibrosarcoma
ARE	Antioxidant response element
UDP	Uridine diphosphate
PGC1	PPARγ coactivator-1
TFAM	Transcription factor A, mitochondrial
EGCG	Epigallocatechin-3-gallate
ULK	Unc-51 like autophagy activating kinase
c-JNK	c-Jun N-terminal kinase
CLEAR	Coordinated lysosomal expression and regulation
HDAC	Histone deacetylase
Atg	Autophagy related
CAMKK	Calcium/Calmodulin-dependent protein kinase kinase
Bcl	Beclin
ERK	Extracellular signal-regulated kinases
HMGCoA	3-hydroxy-3-methyl-glutaryl-Coenzyme A
DNMT	DNA (cytosine-5)-methyltransferase
HO	Heme oxygenase
HSP	Heat shock protein
TNF	Tumor necrosis factor
IL	Interleukin
SOD	Superoxide dismutase
CREB	cAMP response element-binding protein
Bax	Beclin-2- associated X
